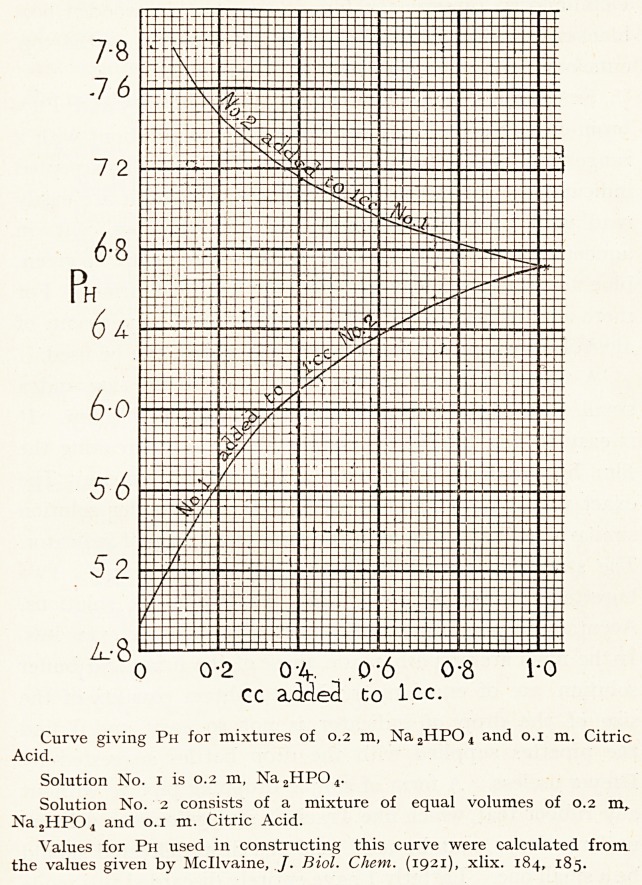# A Film Method for Determining the Reaction of the Liquids of the Body by Indicators

**Published:** 1923-10

**Authors:** George A. Buckmaster

**Affiliations:** Professor of Physiology in the University of Bristol


### The Bristol Medico-Chirurgical Journal

*" Scire est nescire, nisi id me
Scire alius sciret."*

OCTOBER, 1923.

### A FILM METHOD FOR DETERMINING THE REACTION OF THE LIQUIDS OF THE BODY BY INDICATORS.

BY

George A. Buckmaster, M.A., M.D. Oxon.,

*Professor of Physiology in the University of Bristol.*

A large body of evidence has accumulated in support of the electrolytic dissociation theory originally put forward by Arrhenius in 1887, and at the present time the conception of the reaction of a solution, whether acid, alkaline or neutral, is to be regarded either from the point of view of the absolute quantity of neutralisable acid or alkali present, or from the relative concentration of the hydrogen and hydroxyl ions of the solute present in the solution. Therefore in the former case the acidity of a normal solution has been defined as the equivalent of 1 gram atom of acidic hydrogen in 1 litre of solution, and in the latter case a normal solution of hydrogen ion as one containing 1 gram atom of hydrogen ions in a litre of solution. Normal acid contains variable quantities of acid according to the quantity of solution examined, but when ionic hydrogen is the participant in any reaction, the concentration of hydrogen ions is the same in any quantity, great or small, of the solution. A minute amount of liquid possesses the same $H^+$–concentration as the whole of it. On this fact depends the possibility of applying electrometric or colorimetric measurements to drops of liquid. The latter method is dependent upon the former, and in colorimetric determinations we are indirectly using the former.

It is possible to form a conception of the hydrogen ion. It is a colourless substance, unique among other ions, since the element hydrogen is considered to possess only a single electron or unit negative charge to the atom. When this electron is lost by the atom there remains a solitary unit positive charge. This is hydrogen ion $H^+$. All ions move with specific velocities. All ions are hydrated.

All experimental methods possess inherent errors. According to some observers the error with colorimetric determinations for blood is less than with the hydrogen electrode, but except for research, the former method will always remain the one for ordinary use in physiological or clinical laboratories.[1] The colorimetric method for ascertaining the PH of the liquids of the body depends on the use of buffer mixtures which vary in PH, and which have been standardised by hydrogen-electrode measurements. Test-tubes of equal diameter or small bottles with flat polished sides, holding equal volumes of liquid, so arranged

[1] The standard work in English on methods is *The Determination of Hydrogen Ions*, by Mansfield Clark, 2nd edition, 1922. In this will be found full explanations of indicator methods and the reasons for expressing the hydrogen ion concentrations of solutions as PH values; these are, in fact, the negative exponents as whole numbers of the actual hydrogen-ion concentrations. The introduction of PH as a value is due to Sörensen (1909).

in a comparator that standards of known PH can be used for comparison with the liquid, the unknown PH of which can be ascertained by the use of indicators of suitable range, is the usual method of work, but others may be mentioned. Many of the sulphonephthalein group of indicators which give such good results when used in solutions cannot be used for the preparation of stable indicator papers, either sized or unsized, not even if every precaution is taken to keep them in an atmosphere free from carbon dioxide and to protect them from light (A. R. C. Haas). The examination of drops of liquid mixed with drops of indicator upon a highly reflecting surface of opal glass has been suggested by L. D. Felton as a method which is useful for highly-coloured liquids, or where only a little material is available. A feature of this method is the employment of 25 per cent. alcoholic solutions of selected indicators; this facilitates the mixing of the indicator with the drop of buffer solution or solution of which the PH is to be determined. The buffer solutions used were the Clark series, which if kept carefully in glass bottles remain unchanged for two months.

It seemed to me of interest to try whether films of solutions, of urine, plasma, serum, bile or skimmed milk could be used with suitable indicators, and the tints matched with similar films of buffer solutions of known hydrogen-ion concentrations. I found it quite impossible to hold films of these liquids for longer than a minute on rings made of wire. Several kinds of metal, and wire of varying gauge were tried in order to obtain, if possible, durable films. All these attempts were unsuccessful. However, by the use of celloidin rings it is possible to obtain films which will last for half an hour or even an hour, which gives ample time for work. Provided that the area of the ring bears a particular ratio to the breadth of the ring, the formation of a film and its preservation for a long time is possible, indeed quite a simple procedure. I have found that the following dimensions of the celloidin rings give the best results: Diameter of the ring, 14 mm.; opening of the ring, 11 mm.; actual breadth of the ring, 3 mm. The whole ring is carefully cut out from a sheet of celloidin 0.25 mm. thick. It is somewhat larger than a threepenny-piece, and is attached to a handle of celloidin about 6 cm. long. The liquid held in the ring must form a flat film, not a drop. The average weight of a urine film is 0.0154 grammes, and its thickness 0.162 mm. Rings, when not in use, are kept clean by soaking in distilled water. In carrying out this method only indicators in aqueous solutions should be used. Any percentage of alcohol in the indicator over 6 per cent. ruptures the film.

The buffer solution used is the one which has been devised by T. C. McIlvaine.[1] Only two stock solutions are required; 0.2 M (3.56 per cent.) disodium phosphate $Na_2HPO_4 + 2H_2O$, and 0.1 M (2.1 per cent.) citric acid $C_6H_8O_7 + H_2O$. The range of PH extends between 2.2 and 8.0, but a range between 4.9 and 7.8 suffices for the reaction of most of the body liquids. I am indebted to Dr. Millicent Taylor for the construction of the accompanying graph. From this the proportions of the two stock solutions can be at once read off, which will give any PH value desired within the limit range.

The film method lends itself easily to determinations for urine, sweat, bile, saliva, and with certain precautions to milk. The method applied to blood is a special one. It requires smaller celloidin rings, the corpuscles are allowed to gravitate off the film, and the whole procedure must be carried out in an atmosphere with a percentage of carbon dioxide between 5 and 6 per cent.

[1] *J. Biol. Chem.* 1921, xlix., 183.

Curve giving PH for mixtures of 0.2 m, $Na_2HPO_4$ and 0.1 m. Citric Acid.

Solution No. 1 is 0.2 m, $Na_2HPO_4$.

Solution No. 2 consists of a mixture of equal volumes of 0.2 m, $Na_2HPO_4$ and 0.1 m. Citric Acid.

Values for PH used in constructing this curve were calculated from the values given by McIlvaine, *J. Biol. Chem.* (1921), xlix. 184, 185.

Since the method is so easily applicable to urine, the exact procedure of the test with this may be described. Thin celloidin rings are used. They are held horizontal in an easily-constructed apparatus, which consists of a plate of opal glass at an angle of 45° to reflect the light from an electric lamp through the film to the eye. A wooden box, blackened inside, with a hole 5 cm. in diameter at the top, cuts out extraneous light.

Since urines range between an acidity of PH5 and PH7, brom-cresol purple (0.04 per cent. aqueous solution) with a range from yellow (PH5.3) to purple (PH6.5) is the accepted indicator for general use. Differences of 0.2 PH are easily read with practice. Brom-thymol blue (0.4 per cent. in aqueous solution) with a range of PH 6—7.6, yellow to green, blue to blue-green can be used for less acid urines. For more acid urines .02 per cent. methyl-red in 13 per cent. of alcohol, to which a trace of saponin is added, can be used.

A film of urine is picked up on a celloidin ring and a small drop of the indicator allowed to fall on the film. It is easy to mix the liquids completely without breaking the film by gentle shaking with a circular movement. The exact tint is now matched with a standard buffer solution similarly held as a film with the same sized drop of indicator. The standard is made with the help of the graph. Two burettes reading to $\frac{1}{10}$ c.c. hold the two stock solutions. Accuracy is possible to differences of 0.2 PH or even less. If the films are properly made, those of the urine and buffer solution are of equal mass; but to obtain equality of the size of the drop of indicator is not so easy, and I find the pipettes supplied with the drop bottles suggested by Dreyer useless. A form of simple dropping pipette without any rubber teat, which like Traube's stalagmometer delivers uniform drops, may be used, and the drop delivered should be a small one. Latterly I have entirely discarded this mode of adding the indicator, for it is easy to take up an equal quantity of this on another celloidin ring and mix the urine film and indicator film together. Even when the indicators are dichroic, the film, if properly made, is practically without any dichroism.

The following table gives the hydrogen-ion concentrations of various liquids of physiological interest. These values are stated directly in terms of normality (N) and the derived PH notation :—

| | CH | PH |
|---|---|---|
| *Alkalinity.* | | |
| $Na_2HPO_4$, ($\frac{M}{10}$) | $0.000000001 = 1 \times 10^{-9}$ | 9 |
| Pancreatic juice, | $0.00000001 = 1 \times 10^{-8}$ | 8 |
| Blood, | $0.000000038 = .38 \times 10^{-7}$ | 7·4 |
| Blood limits comparable with life | | 7·7—7 |
| Blood variation between arterial and venous blood | | 0.02 |
| *Neutrality.* | | |
| | $0.0000001 = 1 \times 10^{-7}$ | 7 |
| *Acidity.* | | |
| Milk, human, | $1.07 \times 10^{-7}$ | 6.97 |
| Milk, cow, | $2.7 \times 10^{-7}$ | 6.59 |
| Sweat (work), | $6 \times 10^{-7}$ | 6.22 |
| Sweat (heat), | $1.8 \times 10^{-7}$ | 5·73 |
| Urine, | $0.0000001 = 1 \times 10^{-7}$ | 7.0 |
| Urine, | $0.00001 = 1 \times 10^{-5}$ | 5·0 |
| $NaH_2PO_4$, ($\frac{M}{10}$) | $0.0001 = 1 \times 10^{-4}$ | 4 |

## Figures and Tables

**Figure f1:**